# Medical Record Level-Evaluation of Impact of Demographic and Socioeconomic Factors on Pediatric Neuro-Oncology Outcomes at Children’s Hospital Colorado

**DOI:** 10.21203/rs.3.rs-3849043/v1

**Published:** 2024-01-11

**Authors:** Alexandra Pool, Elizabeth Molina Kuna, Amy Anderson-Mellies, Alexandra Kreis, Marcus Marable, Claire Fraley, Daniel Pacheco, Adam L. Green

**Affiliations:** University of Nebraska Medical Center; University of Colorado Cancer Center; University of Colorado Cancer Center; University of Colorado Anschutz Medical Campus; University of Colorado Anschutz Medical Campus; University of Colorado Anschutz Medical Campus; University of Colorado Cancer Center; University of Colorado Anschutz Medical Campus

**Keywords:** pediatric CNS tumors, disparities, survival, treatment, diagnostic timing

## Abstract

**Purpose:**

A medical record-level cohort study to investigate demographic and socioeconomic factors influencing treatment, timing of care, and survival outcomes in pediatric patients diagnosed with central nervous system (CNS) tumors.

**Methods:**

Using electronic health records of patients at Children’s Hospital Colorado from 1986–2020, we identified 898 patients treated for CNS tumors. The primary outcomes of interest were 5-year survival, timing of diagnosis, and treatment. Multivariable logistic regression and Cox regression were used to identify covariates associated with our outcomes of interest.

**Results:**

We found that age, race, tumor type, diagnosis year, and social concerns influenced receipt and timing of treatment. Age, race, patient rural vs. urban residence, and tumor impacted survival outcomes. Time to presentation and treatment were significantly different between White and minority patients. American Indian/Alaska Native and Black patients were less likely to receive chemo compared to White patients (OR 0.28, 0.93 p = 0.037, < 0.001). Patients with 3 + social concerns were more likely to survive after 5 years than children with no or unknown social concerns (OR 1.84, p = 0.011). However, with an adjusted hazards ratio, children with 2 social concerns were less likely to survive to 5 years than children with no or unknown concerns (OR 0.58, p = 0.066).

**Conclusions:**

Demographic and socioeconomic factors influence timing of care and survival outcomes in pediatric patients with CNS tumors. Minority status, age, social factors, rural, and urban patients experience differences in care. This emphasizes the importance of considering these factors and addressing disparities to achieve equitable care.

## Introduction

While great strides in healthcare in the last decades have substantially improved childhood cancer survival, disparities still exist in outcomes between different children based on demographic and socioeconomic factors [1, 2]. Previous cohort studies have identified disparities in the pediatric CNS cancer population leading to differences in outcomes, including survival and extent of disease at presentation, based on race/ethnicity, insurance type, and socioeconomic status, through the use of cancer registries and census information [3]. Specifically, Nieblas-Bedolla *et al*. reviewed 30 studies about disparities in pediatric primary CNS tumors [4]. They found that White and Asian patients were more likely than Hispanic, Black, or Native American patients to be diagnosed with a CNS tumor [4]. However, Hispanic patients were more likely to present with more advanced disease such as metastasis and have worse outcomes. Several studies have found lower survival rates for Black and Hispanic children compared to White children, although not all remained statistically significant with multivariable analysis [5–8]. One study used the SEER database to analyze pediatric patients with brain and CNS tumors form 2000–2015 [9]. This study found that non-Hispanic Whites has a better survival (mean 62 months), while Hispanics, non-Hispanic Blacks, and other racial groups had a poorer survival (mean 40 months) [9].

While these cohort studies have made progress at showing that disparities exist, they have not shown why they exist on an individual patient level, given limitations on clinical information and patient-level socioeconomic status available through cancer registries such as SEER. These studies have suggested that disparities in both pre-diagnostic and post-diagnostic factors exist. In this study, we looked at the individual charts of children diagnosed with a CNS tumor and treated at Children’s Hospital Colorado (CHCO) between the years of 1986 and 2020. It was our goal in this study to more deeply understand factors explaining disparities to better inform social and medical interventions in the future care of such patients. Through our individual chart review methodology, we were able to find personalized information on each patient such as insurance status/provider, specific social concerns expressed to hospital social workers, specific treatment regimens, and diagnostic timing. We hypothesized that we would observe similar disparities to those observed in registry studies and be able to find more specific factors explaining them.

## Methods

### Data Source

Our cohort consisted of pediatric patients diagnosed with CNS tumors between the years 1986 and 2020 and treated at CHCO. This included 1,012 patients, 114 of whom were excluded due to lack of clinical data in their electronic medical record. Most of these patients were patients who had been treated prior to CHCO adopting an EMR and therefore did not have sufficient information in their chart. Others were patients who were seen at CHCO at one point during their care and later lost to follow-up, and therefore did not have enough data in their chart to evaluate.

### Outcomes and Covariates

Our primary outcome of interest was 5-year survival. Other outcomes of interest included receipt of treatment (surgery, chemotherapy, targeted chemotherapy, radiation, and clinical trial treatment), and days from symptom onset to first medical care and diagnosis. Several variables were organized into categorical variables to be adjusted for in the analyses, shown in Table 1. Some covariates were excluded from the final analysis due to lack of statistical or clinical significance, or to eliminate collinearity. The covariates included in our final analyses were patient gender, age, ethnicity, race, urban residence, rural residence, year of diagnosis, tumor type, tumor grade, primary site, metastatic disease, tumor size, and number of social concerns (sum of insurance concerns, financial concerns, and transportation concerns). Covariates were chosen based on having a univariate p-value < 0.1.

### Statistical Analysis

Logistic regression was used to estimate the unadjusted and adjusted odds ratios for receipt of treatment. For our analysis of time from symptom onset to first medical visit and diagnosis, we created a binary variable using the median number of days to care to use as a threshold in a binary logistic regression analysis to examine variables associated with more or less time to care. To estimate survival, Kaplan-Meier methods and the log-rank test were used to compare survival across various strata. We then used Cox proportional hazards models to estimate unadjusted and adjusted hazard ratios. All analyses were completed in SAS version 9.4 (SAS Inc., Cary NC) and evaluated as significant at p < 0.1, given the single-institution nature and relatively small size of the cohort.

## Results

Our final cohort included 898 pediatric patients diagnosed with CNS tumors during our study period. Table 1 shows the cohort characteristics of patients by demographic, socioeconomic, and clinical variables.

### Treatment

Treatment characteristics by demographic, socioeconomic, and clinical variables are shown in Table 2.

The univariate treatment analysis showed that female patients were less likely to receive any chemotherapy (OR 0.74, p = 0.022) and cytotoxic chemotherapy (OR 0.68, p = 0.006) than male patients (Table 2). American Indian and Alaskan Native patients were more likely to receive radiation therapy than white patients (OR 2.35, p-value = 0.061). Asian and Pacific Islander patients were less likely to be enrolled in a clinical trial than white patients (OR 0.48, p-value = 0.095). Black children were also less likely than white to receive any type of chemotherapy (OR 0.93 p-value < 0.001). Children from outside the CHCO region were more likely to receive any type of chemotherapy when compared with children within 12.5 miles of CHCO (OR 2.61, p = 0.012). Rural patients were more likely to receive targeted chemotherapy when compared with urban patients (OR 1.41, p = 0.094). Patients with insurance concerns, financial concerns and transport concerns were more likely than patients without such concerns to receive radiation treatment (OR 1.80, 2.60, 2.23 p-values 0.005, < 0.001, 0.005) and chemotherapy (Table 5). Patients with financial concerns were also more likely to be enrolled in a clinical trial than patients without financial concerns (OR 1.66, p-value = 0.002). Diagnosis year, tumor type, grade, metastatic disease status, and tumor size at diagnosis were all associated with differences in treatment, as expected.

Our next step was to perform a multivariable analysis comparing treatment across categories of patients (Table 3). In terms of rural vs. urban residence, patients with unknown status were less likely than rural patients to receive surgery, radiation therapy, general chemotherapy, or cytotoxic chemotherapy. American Indian/Alaska Native patients were less likely than White patients to receive any chemotherapy or targeted chemotherapy (OR 0.28, 0.13 p = 0.037, 0.023). Patients with one social concern were more likely to receive radiation and targeted chemotherapy when compared to patients with no/unknown social concerns. Patients with three or more social concerns were more likely to receive any form of chemotherapy but were more likely to receive radiation or be enrolled in a clinical trial than patients with no/unknown social concerns. In general, patients with more aggressive diagnoses, such as high-grade glioma or medulloblastoma, were more likely to receive each treatment than patients diagnosed with less aggressive disease, such as low-grade glioma. Older children were more likely to receive surgery and radiation than younger age groups. 1–4 year-old patients were the most likely to receive any form of chemotherapy and to be enrolled in a clinical trial. Year of diagnosis played a role in what treatment patients received, with patients diagnosed 2001–2010 more likely to receive surgery. Patients diagnosed before 2010 were more likely to receive cytotoxic chemotherapy and be enrolled in a clinical trial, but less likely to receive targeted chemotherapy. Metastatic disease status also affected which patients received different treatments, as expected.

### Diagnostic Timing

We examined the timing from the onset of symptoms to first medical care in our patients for whom we had diagnostic timing data. Due to known differences in timing of symptoms based on disease type, we ran separate analyses for children with low-grade (N = 237) and high-grade (N = 183) tumors. To compare the time from first symptoms to care, the median number of days from symptom onset to first medical care was used as the threshold; 54 days was the median time among low-grade tumors, and 31 days was the median for those with high-grade tumors. Among low-grade tumor patients, children 5–9 years old were more likely than 1–4-year-old children to receive care within 54 days of symptom onset (OR 2.96, p = 0.006, Table 4). Likewise, Black children with low-grade tumors were more likely than White children to receive care within 54 days of symptom onset (OR 8.56, p = 0.047). Children with low-grade tumors and 3 or more social concerns were also more likely than children with no/unknown social concerns to receive care within 54 days of symptom onset (OR 2.62, p = 0.033). Among those with high-grade tumors, children 5–9 or 15 + years old were less likely to receive care within 31 days of symptom onset than children 1–4 years old (OR 0.28, 0.18, p = 0.004, < 0.001). Hispanic children were less likely than non-Hispanic children with a high-grade tumor to receive care within 31 days of symptom onset (OR 0.52, p = 0.046). Children diagnosed with a high grade tumor between 2001 and 2010 were more likely to get care within 31 days than children diagnosed between 2011–2020 (OR 2.02, p-value = 0.072).

We also examined the timing between first receipt of care for symptoms to the time of diagnosis. Analyses were again separated by low- (N = 341) and high-grade (N = 221) tumors, and the median number of days was used as the threshold (Table 5). Children 15 + years old with a low-grade tumor were less likely than children 1–4 years old to receive a diagnosis within 5 days from their first care encounter (OR 0.52, p = 0.097). Black children with a low-grade tumor were less likely than white children to receive a diagnosis in less than 5 days from first care (OR 0.15, p = 0.069). Patients with low-grade tumors greater than 6 cm in size were more likely than those with smaller tumors to take less than 5 days to receive a diagnosis from first care (OR 3.59, p = 0.015). Additionally, children with low-grade tumors and one social concern were less likely to receive a diagnosis within 5 days than children with no/unknown social concerns (OR 0.57, p = 0.082). Among those with high-grade tumors, children from rural settings were more likely to receive a diagnosis less than 3 days after presenting than children from an urban setting (OR 1.85, p = 0.055). Finally, children diagnosed 2001–2010 with a high-grade tumor were more likely to have less than 3 days between care and diagnosis than children diagnosed between 2011 and 2020 (OR 1.99, p = 0.020).

On multivariable analysis of diagnostic timing, those 5–9 years old with low-grade tumors were more likely than children 1–4 years old with a low-grade tumor to receive care within 54 days of symptom onset (OR 3.36, p = 0.005 Table 4). Black children with low-grade tumors were more likely than white children with-low-grade tumors to receive care within 54 days of symptom onset (OR 6.63, p = 0.097). Children with 3 or more social concerns were more likely than children with a low-grade tumor and no/unknown social concerns to receive care within 54 days of symptom onset (OR 2.71, p = 0.059). Among high-grade tumor patients, children 5–9 or 15 + years old were less likely than children 1–4 years old with a high-grade tumor to receive care within 31 days of symptom onset (OR 0.31, 0.17, p = 0.018, 0.001). Children diagnosed between the years 2001 and 2010 with a high-grade tumor were more likely to have care within 31 days of symptom onset than children diagnosed between 2011 and 2020 (OR 2.01, p = 0.072).

Among those with a low-grade tumor, children 5–9 years old or 15 and older were less likely to receive a diagnosis in less than 5 days from presentation than children 1–4 years old (OR 0.51, 0.46, p = 0.065, 0.079, Table 5). Children diagnosed before 2001 with a low-grade tumor were more likely to receive a diagnosis less than 5 days after first receiving care than children diagnosed in 2011–2020 (OR 7.47, p < 0.0001). Children with a low-grade tumor 6 centimeters in size or greater were more likely to receive a diagnosis less than 5 days after first receiving care than children with smaller tumors (OR 4.31, p = 0.011). In addition, children with a high-grade tumor diagnosed in the years 2001–2010 were more likely to receive a diagnosis in less than 3 days after first receiving care than children diagnosed with a high-grade tumor in 2011–2020 (OR 2.08, p = 0.027).

### Survival:

Finally, we evaluated factors associated with overall survival in all patients, excluding those whose chart did not have a vital status or follow-up time noted; 858 patients were included in the survival analysis. Children 10–14 or 5–9 years old had better survival outcomes than children 1–4 years old (HR 0.48, 0.55, p = 0.012, 0.014, Table 5). Children living in rural areas had worse survival outcomes than those in urban areas (HR 1.40, p = 0.092). Patients with high-grade tumors with a primary site in the brainstem or with metastatic disease were less likely to survive than their comparison groups. Children with 3 or more social concerns were less likely to survive than children with no/unknown social concerns (HR 1.84, p = 0.011).

When adjusting for our covariates of interest, it was found that Asian/Pacific Islander patients had significantly worse survival outcomes than White patients (HR 3.59, CI 1.32–9.76, p = 0.012). Patients diagnosed between 2001–2010 were more likely to survive than patients diagnosed between 2011–2022.

## Discussion

This study aimed to investigate the factors influencing treatment, timing of care, and survival outcomes in pediatric patients diagnosed with CNS tumors at a single institution through deep chart review. The findings highlight several important considerations in the management of these patients and provide insights into potential areas for improvement in their care.

Geographic factors, such as rural and urban residence, were associated with differences in treatment modalities and timing of care. Rural patients were more likely to receive chemotherapy, highlighting potential variations in treatment practices between different patient demographics. Rural patients were also more likely to receive a diagnosis in 3 days after presenting to the hospital for treatment. Previous studies have established that there are differences in disease prevalence between rural and urban populations as well as different race/ethnicity subpopulations within rural and urban areas [10–11]. In general, there are fewer barriers to care for urban populations when compared with rural populations. For example, urban populations are more likely to have large hospitals and specialist access, whereas rural patients often need to travel hours to access this specialty care [11, 12]. Our study found that the difference between these populations is present in the treatment received as well as timing of diagnosis.

Race and ethnicity also played a role in disparities, as black patients were less likely to any chemotherapy treatments and Asian and Pacific islander patients were less likely to be enrolled in clinical trials, which has been noted in other studies as well [12–17]. Black patients with low-grade tumors tended to receive medical care sooner but have a longer time from presentation to diagnosis than White children. Hispanic children with high-grade tumors were also more likely to receive care sooner, with no difference in time to diagnosis. Other studies have shown that Black patients with certain cancers such as soft tissue sarcomas present at a higher stage than White patients and have worse outcomes [18]. Hispanic children with retinoblastomas have on average shorter survival and greater invasion of disease than White children, and all minority groups with retinoblastoma are more likely to present with extra-ocular disease [19, 20]. Identifying and addressing barriers that may disproportionately affect non-White patients is essential to promote equitable care and decrease severity of disease at presentation.

Survival outcomes were influenced by various factors, including age, race, tumor grade, and social concerns. The finding of worse survival outcomes among Asian/Pacific Islander patients compared to White patients has been observed before and highlights the need for further investigation into potential contributing factors and tailored interventions to improve outcomes for this population [21, 12]. Unlike previous studies, our data did not show a survival difference between other non-White patients and White patients [22]. CHCO and Denver’s safety net hospital, Denver Health, may be protective of traditionally underserved populations in this case.

These medical resources in our region may also explain why increased social concerns in our study were not associated with negative outcomes on adjusted analysis; indeed, patients with three or more social concerns were actually more likely to present to care sooner with low-grade tumor symptoms than patients with no/unknown social concerns. This finding runs contrary to previous work finding that patients with lower socioeconomic status tend to present later in their disease course [22]. Social concerns also played a role in treatment disparities. Patients with social concerns were more likely to receive certain treatments, e.g. radiation and chemotherapy, than patients with no/unknown social concerns. Other studies had previously established that lower SES patients have poorer survival outcomes especially with ALL and AML [22]; while patients with increased social concerns had poorer survival on univariate analysis in our study, this was not the case when adjusting for other factors.

This study is limited by the retrospective nature of the study and the use of data from a single institution, which may limit the generalizability of the findings. Future research should aim to validate these findings in larger, multicenter studies to enhance the generalizability of the results.

## Figures and Tables

**Table 1:** Cohort Characteristics. This table lists the number (n) and percentage (%) of patients matching each characteristic on chart review.

Variable	Surgery, n	Surgery, %	Radiation, n	Radiation, %	Clinical trial, n	Clinical trial, %	Chemotherapy (any), n	Chemotherapy (any), %	Chemotherapy (cytotoxic), n	Chemotherapy (cytotoxic), %	Chemotherapy (targeted), n	Chemotherapy (targeted), %
**Gender**												
Male	344	72.27	197	41.39	225	47.27	258	54.20	191	40.13	82	17.23
Female	291	69.45	158	37.71	198	47.26	195	46.54	131	31.26	77	18.38
Unknown	1	33.33	1	33.33	0	0.00	2	66.67	2	66.67	1	33.33
**Age**												
< 1 year	34	66.67	13	25.49	22	43.14	29	56.86	23	45.10	5	9.80
1–4 years	164	71.62	97	42.36	131	57.21	142	62.01	104	45.41	53	23.14
5–9 years	163	67.92	82	34.17	108	45.00	106	44.17	70	29.17	40	16.67
10–14 years	151	72.95	80	38.65	93	44.93	90	43.48	65	31.40	32	15.46
15 + years	110	74.83	72	48.98	61	41.50	74	50.34	56	38.10	25	17.01
Unknown	14	58.33	12	50.00	8	33.33	14	58.33	6	25.00	5	20.83
**Ethnicity**												
Hispanic	134	74.86	81	45.25	92	51.40	102	56.98	78	43.58	36	20.11
Non-Hispanic	420	71.79	239	40.85	302	51.62	310	52.99	216	36.92	112	19.15
Unknown	82	61.19	36	26.87	29	21.64	43	32.09	30	22.39	12	8.96
**Race**												
White	471	72.35	266	40.86	344	52.84	357	54.84	254	39.02	123	18.89
Black	24	70.59	12	35.29	18	52.94	18	52.94	11	32.35	7	20.59
AI/AN	18	85.71	13	61.90	12	57.14	12	57.14	11	52.38	2	9.52
Asian/PI	13	56.52	11	47.83	8	34.78	12	52.17	10	43.48	5	21.74
Unknown	110	65.09	54	31.95	41	24.26	56	33.14	38	22.49	23	13.61
**Primary Language**												
English	416	71.48	256	43.99	263	45.19	305	52.41	214	36.77	135	23.20
Spanish	29	78.38	20	54.05	14	37.84	23	62.16	17	45.95	9	24.32
Unknown	191	68.46	80	28.67	146	52.33	127	45.52	93	33.33	16	5.73
**Insurance at first surgery**												
Private	246	84.83	131	45.17	162	55.86	152	52.41	104	35.86	65	22.41
Public/Uninsured	175	82.94	98	46.45	109	51.66	117	55.45	85	40.28	39	18.48
Unknown	215	54.16	127	31.99	152	38.29	186	46.85	135	34.01	56	14.11
**Distance from CHCO**												
< 2.5 miles	128	72.73	74	42.05	88	50.00	90	51.14	67	38.07	34	19.32
2.5 to 50 miles	180	72.29	99	39.76	120	48.19	125	50.20	91	36.55	40	16.06
> 50 to 300 miles	218	72.67	109	36.33	139	46.33	155	51.67	106	35.33	58	19.33
> 300 miles	71	70.30	49	48.51	42	41.58	51	50.50	36	35.64	17	16.83
Outside of CHCO region	29	70.73	21	51.22	25	60.98	30	73.17	21	51.22	11	26.83
Unknown	10	32.26	4	12.90	9	29.03	4	12.90	3	9.68	0	0.00
**Urban/Rural**												
Urban	495	72.05	282	41.05	330	48.03	358	52.11	257	37.41	119	17.32
Rural	131	72.78	70	38.89	84	46.67	93	51.67	64	35.56	41	22.78
Unknown	10	32.26	4	12.90	9	29.03	4	12.90	3	9.68	0	0.00
**Diagnosis Year**												
Before 2001	59	58.42	21	20.79	36	35.64	41	40.59	34	33.66	2	1.98
2001–2010	191	72.62	91	34.60	162	61.60	135	51.33	98	37.26	31	11.79
2011–2020	373	72.85	232	45.31	217	42.38	265	51.76	186	36.33	122	23.83
Unknown	13	59.09	12	54.55	8	36.36	14	63.64	6	27.27	5	22.73
**Tumor Type**												
Low-grade glioma	308	71.96	67	15.65	158	36.92	164	38.32	114	26.64	69	16.12
High-grade glioma	48	54.55	60	68.18	45	51.14	56	63.64	29	32.95	40	45.45
Other/unspecified glioma	11	33.33	7	21.21	7	21.21	8	24.24	3	9.09	3	9.09
Embryonal tumor	18	69.23	22	84.62	23	88.46	25	96.15	20	76.92	3	11.54
Ependymoma	49	90.74	41	75.93	38	70.37	26	48.15	18	33.33	14	25.93
Germ cell tumors	21	45.65	32	69.57	24	52.17	40	86.96	33	71.74	1	2.17
Craniopharyngioma	35	97.22	21	58.33	18	50.00	10	27.78	2	5.56	10	27.78
ATRT	22	100.00	17	77.27	19	86.36	20	90.91	16	72.73	0	0.00
Meningioma	10	83.33	6	50.00	3	25.00	4	33.33	4	33.33	0	0.00
Medulloblastoma	71	91.03	66	84.62	62	79.49	73	93.59	64	82.05	8	10.26
Choroid plexus	14	77.78	2	11.11	2	11.11	3	16.67	3	16.67	1	5.56
Other/Unknown	29	50.88	15	26.32	24	42.11	26	45.61	18	31.58	11	19.30
**Grade**												
Low	109	82.58	23	17.42	28	21.21	47	35.61	30	22.73	30	22.73
High	75	84.27	77	86.52	59	66.29	78	87.64	58	65.17	33	37.08
Unknown	452	66.77	256	37.81	336	49.63	330	48.74	236	34.86	97	14.33
**Metastatic**												
Present	43	81.13	35	66.04	37	69.81	45	84.91	38	71.70	14	26.42
Absent	238	83.51	144	50.53	147	51.58	176	61.75	122	42.81	71	24.91
Unknown	355	63.39	177	31.61	239	42.68	234	41.79	164	29.29	75	13.39
**Tumor Size**												
Less than 6cm	122	78.71	77	49.68	83	53.55	83	53.55	58	37.42	32	20.65
6cm or greater	45	91.84	29	59.18	26	53.06	30	61.22	23	46.94	14	28.57
Unknown	469	67.58	250	36.02	314	45.24	342	49.28	243	35.01	114	16.43
**Primary Site**												
Brainstem	34	47.89	34	47.89	34	47.89	38	53.52	19	26.76	19	26.76
Hemispheres	155	77.89	67	33.67	83	41.71	78	39.20	52	26.13	34	17.09
Meningeal	8	80.00	5	50.00	4	40.00	4	40.00	3	30.00	1	10.00
Non-CNS	3	75.00	2	50.00	3	75.00	3	75.00	2	50.00	2	50.00
Other CNS	21	67.74	7	22.58	12	38.71	15	48.39	13	41.94	4	12.90
PNS	3	60.00	2	40.00	2	40.00	2	40.00	1	20.00	0	0.00
Pineal	24	54.55	32	72.73	31	70.45	40	90.91	32	72.73	0	0.00
Posterior fossa	195	85.90	99	43.61	121	53.30	111	48.90	88	38.77	29	12.78
Sellar/Suprasellar	75	52.08	38	26.39	61	42.36	85	59.03	54	37.50	43	29.86
Spinal cord	35	79.55	15	34.09	17	38.64	17	38.64	13	29.55	4	9.09
Thalamus	30	71.43	23	54.76	25	59.52	22	52.38	15	35.71	10	23.81
Unknown	6	60.00	5	50.00	8	80.00	7	70.00	6	60.00	2	20.00
Ventricles	47	70.15	27	40.30	22	32.84	33	49.25	26	38.81	12	17.91
**Number of Social Concerns**												
One	130	72.63	86	48.04	86	48.04	100	55.87	65	36.31	44	24.58
Two	105	77.21	69	50.74	69	50.74	75	55.15	60	44.12	25	18.38
3 or more	80	79.21	54	53.47	59	58.42	71	70.30	53	52.48	32	31.68
None/Unknown	321	66.60	147	30.50	209	43.36	209	43.36	146	30.29	59	12.24
**Insurance concerns**												
Yes	80	74.77	56	52.34	56	52.34	64	59.81	48	44.86	27	25.23
No/Unknown	556	70.29	300	37.93	367	46.40	391	49.43	276	34.89	133	16.81
**Financial concerns**												
Yes	134	74.44	105	58.33	103	57.22	126	70.00	91	50.56	57	31.67
No/Unknown	502	69.92	251	34.96	320	44.57	329	45.82	233	32.45	103	14.35
**Transportation concerns**												
Yes	43	78.18	32	58.18	28	50.91	35	63.64	28	50.91	12	21.82
No/Unknown	593	70.34	324	38.43	395	46.86	420	49.82	296	35.11	148	17.56

**Table 2:** Univariate analysis of receipt of treatment. This table looks at the odds ratio with a 90% confidence interval and p-value of receiving surgery, radiation, enrollment in a clinical trial, any form of chemotherapy, cytotoxic chemotherapy, or targeted chemotherapy. Significant p-values are bolded.

		Surgery		Radiation		Clinical Trial		Chemo (any)		Chemo (cytotoxic)		Chemo (targeted)	
Variable		OR (90% CI)	p-value	OR (90% CI)	p-value	OR (90% CI)	p-value	OR (90% CI)	p-value	OR (90% CI)	p-value	OR (90% CI)	p-value
**Gender**	Male	*ref*		*ref*		*ref*		*ref*		*ref*		*ref*	
	Female	0.87 (0.68, 1.11)	0.354	0.86 (0.68, 1.07)	0.262	1.00 (0.80, 1.25)	0.997	0.74 (0.59, 0.92)	**0.022**	0.68 (0.54, 0.86)	**0.006**	1.08 (0.81, 1.44)	0.653
	Unknown	0.19 (0.03, 1.45)	0.179	0.71 (0.09, 5.34)	0.779	--	.	1.69 (0.22, 12.74)	0.669	2.98 (0.40, 22.49)	0.374	2.40 (0.32, 18.19)	0.476
**Age**	< 1 year	0.79 (0.46, 1.37)	0.483	0.47 (0.26, 0.83)	**0.028**	0.57 (0.34, 0.95)	**0.070**	0.81 (0.48, 1.35)	0.496	0.99 (0.59, 1.65)	0.967	0.36 (0.16, 0.82)	**0.040**
	1–4 years	*ref*		*ref*		*ref*		*ref*		*ref*		*ref*	
	5–9 years	0.84 (0.60, 1.17)	0.384	0.71 (0.52, 0.97)	0.068	0.61 (0.45, 0.83)	**0.008**	0.48 (0.36, 0.66)	**< .001**	0.49 (0.36, 0.68)	**< .001**	0.66 (0.45, 0.98)	**0.080**
	10–14 years	1.07 (0.75, 1.52)	0.757	0.86 (0.62, 1.18)	0.431	0.61 (0.44, 0.84)	**0.011**	0.47 (0.34, 0.65)	**< .001**	0.55 (0.40, 0.76)	**0.003**	0.61 (0.40, 0.91)	**0.044**
	15–19 years	1.18 (0.79, 1.75)	0.377	1.31 (0.92, 1.85)	0.208	0.53 (0.37, 0.75)	**0.003**	0.62 (0.44, 0.88)	**0.026**	0.74 (0.52, 1.05)	0.162	0.68 (0.44, 1.06)	0.154
**Ethnicity**	Non-Hispanic	*ref*		*ref*		*ref*		*ref*		*ref*		*ref*	
	Hispanic	1.17 (0.85, 1.61)	0.422	1.20 (0.90, 1.59)	0.297	0.99 (0.75, 1.31)	0.958	1.18 (0.89, 1.56)	0.349	1.32 (0.99, 1.75)	0.110	1.06 (0.75, 1.51)	0.775
	Unknown	0.62 (0.45, 0.86)	**0.016**	0.53 (0.38, 0.75)	**0.003**	0.26 (0.18, 0.38)	**< .001**	0.42 (0.30, 0.59)	**< .001**	0.49 (0.34, 0.71)	**0.002**	0.42 (0.25, 0.70)	**0.006**
**Race**	White	*ref*		*ref*		*ref*		*ref*		*ref*		*ref*	
	Black	0.92 (0.49, 1.73)	0.823	0.79 (0.43, 1.45)	0.520	1.00 (0.56, 1.79)	0.991	0.93 (0.52, 0.70)	**< .001**	0.75 (0.40, 1.39)	0.438	1.11 (0.54, 2.28)	0.806
	AI/AN	2.23 (0.81, 6.46)	0.188	2.35 (1.11, 4.98)	**0.061**	1.19 (0.57, 2.49)	0.698	1.10 (0.53, 2.29)	0.835	1.72 (0.83, 3.57)	0.223	0.45 (0.13, 1.55)	0.290
	Asian/ PI	0.50 (0.25, 1.01)	0.103	1.33 (0.66, 2.67)	0.506	0.48 (0.23, 0.99)	**0.095**	0.90 (0.45, 1.81)	0.801	1.20 (0.59, 2.43)	0.667	1.19 (0.51, 2.78)	0.733
Unknown	0.71 (0.53, 0.96)	**0.065**	0.68 (0.50, 0.92)	**0.035**	0.29 (0.21, 0.39)	**< .001**	0.41 (0.30, 0.55)	**< .001**	0.45 (0.33, 0.63)	**< .001**	0.68 (0.45, 1.01)	0.111
**Primary Language**	English	*ref*		*ref*		*ref*		*ref*		*ref*		*ref*	
	Spanish	1.45 (0.74, 2.84)	0.368	1.50 (0.86, 2.62)	0.235	0.74 (0.42, 1.31)	0.385	1.49 (0.84, 2.65)	0.252	1.46 (0.83, 2.56)	0.265	1.06 (0.56, 2.04)	0.875
	Unknown	0.87 (0.64, 1.19)	0.391	0.51 (0.40, 0.66)	**< .001**	1.33 (1.05, 1.69)	**0.035**	0.76 (0.60, 0.96)	**0.059**	0.86 (0.67, 1.11)	0.325	0.20 (0.13, 0.32)	**< .001**
**Insurance at first surgery**	Private	*ref*		*ref*		*ref*		*ref*		*ref*		*ref*	
	Public/Uninsured	0.87 (0.58, 1.30)	0.569	1.05 (0.78, 1.42)	0.778	0.84 (0.63, 1.14)	0.351	1.13 (0.84, 1.52)	0.501	1.21 (0.89, 1.64)	0.314	0.78 (0.54, 1.14)	0.284
	Unknown	0.21 (0.15, 0.29)	**< .001**	0.57 (0.44, 0.74)	**< .001**	0.49 (0.38, 0.63)	**< .001**	0.80 (0.62, 1.03)	0.150	0.92 (0.71, 1.20)	0.614	0.57 (0.41, 0.79)	**0.005**
**Distance from CHCO**	< 12.5 miles	*ref*		*ref*		*ref*		*ref*		*ref*		*ref*	
	12.5 to 50 miles	0.98 (0.68, 1.41)	0.921	0.91 (0.65, 1.26)	0.637	0.93 (0.67, 1.29)	0.714	0.96 (0.70, 1.33)	0.849	0.94 (0.67, 1.31)	0.749	0.80 (0.52, 1.22)	0.384
	> 50 to 300 miles	1.00 (0.70, 1.42)	0.989	0.79 (0.57, 1.08)	0.217	0.86 (0.63, 1.18)	0.440	1.02 (0.75, 1.40)	0.911	0.89 (0.64, 1.23)	0.549	1.00 (0.67, 1.49)	0.997
	> 300 miles	0.89 (0.56, 1.40)	0.665	1.30 (0.86, 1.96)	0.297	0.71 (0.47, 1.08)	0.177	0.97 (0.65, 1.47)	0.918	0.90 (0.59, 1.38)	0.688	0.85 (0.49, 1.45)	0.608
	Outside of CHCO region	0.91 (0.48, 1.70)	0.797	1.45 (0.82, 2.56)	0.288	1.56 (0.87, 2.80)	0.207	2.61 (1.39, 4.90)	**0.012**	1.71 (0.96, 3.03)	0.125	1.53 (0.79, 2.96)	0.288
	Unknown	0.21 (0.15, 0.29)	**< .001**	0.20 (0.08, 0.51)	**0.004**	0.41 (0.20, 0.82)	**0.035**	0.14 (0.06, 0.35)	**< .001**	0.17 (0.06, 0.49)	**0.005**	--	.
**Urban/Rural**	Urban	*ref*		*ref*		*ref*		*ref*		*ref*		*ref*	
	Rural	1.04 (0.76, 1.41)	0.847	0.91 (0.69, 1.21)	0.600	0.95 (0.72, 1.25)	0.744	0.98 (0.75, 1.29)	0.915	0.92 (0.69, 1.23)	0.647	1.41 (1.01, 1.97)	**0.094**
	Unknown	0.18 (0.10, 0.35)	**< .001**	0.21 (0.09, 0.52)	**0.004**	0.44 (0.23, 0.86)	**0.043**	0.14 (0.06, 0.33)	**< .001**	0.18 (0.07, 0.49)	**0.005**	--	.
**Year of Diagnosis**	Before 2001	0.52 (0.36, 0.76)	**0.004**	0.32 (0.21, 0.49)	**< .001**	0.75 (0.52, 1.09)	0.210	0.64 (0.44, 0.92)	**0.041**	0.89 (0.61, 1.30)	0.610	0.06 (0.02, 0.21)	**< .001**
	2001–2010	0.99 (0.75, 1.31)	0.946	0.64 (0.49, 0.83)	**0.004**	2.18 (1.69, 2.81)	**< .001**	0.98 (0.77, 1.26)	0.910	1.04 (0.80, 1.35)	0.798	0.43 (0.30, 0.61)	**< .001**
	2011–2020	*ref*		*ref*		*ref*		*ref*		*ref*		*ref*	
	Unknown	0.54 (0.26, 1.12)	0.164	1.45 (0.71, 2.97)	0.397	0.78 (0.37, 1.63)	0.577	1.63 (0.78, 3.43)	0.279	0.66 (0.29, 1.47)	0.389	0.94 (0.40, 2.21)	0.905
**Tumor Type**	Low-grade glioma	*ref*		*ref*		*ref*		*ref*		*ref*		*ref*	
	High-grade glioma	0.47 (0.32, 0.69)	**0.002**	11.55 (7.47, 17.85)	**< .001**	1.79 (1.21, 2.63)	**0.014**	2.82 (1.89, 4.20)	**< .001**	1.35 (0.89, 2.05)	0.229	4.34 (2.87, 6.55)	**< .001**
	Other/unspecified glioma	0.19 (0.10, 0.37)	**< .001**	1.45 (0.70, 3.02)	0.404	0.46 (0.22, 0.94)	0.076	0.52 (0.26, 1.02)	0.113	0.28 (0.10, 0.76)	**0.036**	0.52 (0.19, 1.44)	0.292
	Embryonal tumor	0.88 (0.43, 1.80)	0.764	29.63 (11.80, 74.40)	**< .001**	13.10 (4.71, 36.44)	**< .001**	40.24 (7.46, 217.09)	**< .001**	9.18 (4.18, 20.16)	**< .001**	0.68 (0.24, 1.91)	0.537
	Ependymoma	3.82 (1.73, 8.43)	**0.005**	16.99 (9.63, 29.97)	**< .001**	4.06 (2.42, 6.81)	**< .001**	1.49 (0.93, 2.41)	0.166	1.38 (0.83, 2.29)	0.300	1.82 (1.05, 3.17)	**0.075**
	Germ cell tumors	0.33 (0.19, 0.55)	**< .001**	12.32 (6.96, 21.79)	**< .001**	1.86 (1.12, 3.11)	**0.046**	10.73 (5.13, 22.46)	**< .001**	6.99 (3.96, 12.34)	**< .001**	0.12 (0.02, 0.62)	**0.034**
	Craniopharyngioma	13.64 (2.55, 72.99)	**0.010**	7.54 (4.15, 13.71)	**< .001**	1.71 (0.96, 3.03)	0.124	0.62 (0.33, 1.17)	0.213	0.16 (0.05, 0.54)	**0.013**	2.00 (1.05, 3.83)	**0.079**
	ATRT	2000045 (0.00, ∞)	0.976	18.32 (7.71, 43.51)	**< .001**	10.82 (3.84, 30.47)	**< .001**	16.10 (4.70, 55.12)	**< .001**	7.35 (3.28, 16.47)	**< .001**	--	.
	Meningioma	1.95 (0.54, 7.05)	0.394	5.39 (2.03, 14.28)	**0.004**	0.57 (0.19, 1.73)	0.404	0.80 (0.29, 2.23)	0.726	1.38 (0.50, 3.83)	0.607	--	.
	Medulloblastoma	3.95 (2.01, 7.76)	**< .001**	29.63 (16.92, 51.91)	**<.001**	6.62 (4.06, 10.81)	**< .001**	23.50 (10.80, 51.16)	**< .001**	12.59 (7.50, 21.13)	**< .001**	0.59 (0.31, 1.14)	0.189
	Choroid plexus	1.36 (0.53, 3.52)	0.591	0.67 (0.19, 2.36)	0.604	0.21 (0.06, 0.74)	**0.041**	0.32 (0.11, 0.92)	0.077	0.55 (0.19, 1.58)	0.353	0.31 (0.06, 1.69)	0.254
	Unknown	0.40 (0.25, 0.65)	**0.002**	1.92 (1.12, 3.31)	**0.047**	1.24 (0.78, 1.99)	0.448	1.35 (0.85, 2.15)	0.290	1.27 (0.77, 2.10)	0.432	1.24 (0.69, 2.25)	0.544
**Grade**	Low	*ref*		*ref*		*ref*		*ref*		*ref*		*ref*	
	High	1.13 (0.61, 2.08)	0.741	30.40 (16.11, 57.35)	**< .001**	7.30 (4.39, 12.15)	**< .001**	12.82 (6.98, 23.56)	**< .001**	6.36 (3.86, 10.49)	**< .001**	2.00 (1.22, 3.29)	**0.021**
	Unknown	0.42 (0.28, 0.63)	**< .001**	2.88 (1.93, 4.30)	**< .001**	3.66 (2.52, 5.31)	**< .001**	1.72 (1.24, 2.38)	**0.006**	1.82 (1.26, 2.62)	**0.007**	0.57 (0.39, 0.84)	**0.016**
**Metastatic Disease**	Present	0.85 (0.45, 1.60)	0.672	1.90 (1.14, 3.19)	**0.04**	2.17 (1.28, 3.68)	**0.016**	3.48 (1.80, 6.75)	**0.002**	3.38 (1.97, 5.80)	**< .001**	1.08 (0.62, 1.89)	0.817
	Absent	*ref*		*ref*		*ref*		*ref*		*ref*		*ref*	
	Unknown	0.34 (0.25, 0.46)	**< .001**	0.45 (0.35, 0.58)	**< .001**	0.70 (0.55, 0.89)	**0.014**	0.44 (0.35, 0.57)	**< .001**	0.55 (0.43, 0.71)	**< .001**	0.47 (0.34, 0.63)	**< .001**
**Tumor Size**	Less than 6cm	*ref*		*ref*		*ref*		*ref*		*ref*		*ref*	
	6cm or greater	3.04 (1.22, 7.61)	**0.046**	1.47 (0.85, 2.54)	0.247	0.98 (0.57, 1.68)	0.952	1.37 (0.79, 2.37)	0.347	1.48 (0.86, 2.55)	0.237	1.54 (0.83, 2.84)	0.249
	Unknown	0.56 (0.40, 0.80)	**0.007**	0.57 (0.42, 0.77)	**0.002**	0.72 (0.53, 0.96)	**0.062**	0.84 (0.63, 1.13)	0.337	0.90 (0.67, 1.22)	0.572	0.76 (0.52, 1.09)	0.209
**Insurance Concerns**	Yes	1.25 (0.85, 1.85)	0.340	1.80 (1.28, 2.53)	**0.005**	1.27 (0.90, 1.78)	0.249	1.52 (1.08, 2.15)	**0.045**	1.52 (1.08, 2.14)	**0.045**	1.67 (1.12, 2.49)	**0.034**
	No/Unknown	*ref*		*ref*		*ref*		*ref*		*ref*		*ref*	
**Financial Concerns**	Yes	1.25 (0.92, 1.71)	0.233	2.60 (1.97, 3.45)	**< .001**	1.66 (1.26, 2.19)	**0.002**	2.76 (2.06, 3.70)	**< .001**	2.13 (1.61, 2.81)	**< .001**	2.77 (2.02, 3.80)	**< .001**
	No/Unknown	*ref*		*ref*		*ref*		*ref*		*ref*		*ref*	
**Transportation Concerns**	Yes	1.51 (0.87, 2.62)	0.219	2.23 (1.40, 3.55)	**0.005**	1.18 (0.74, 1.86)	0.559	1.76 (1.10, 2.83)	**0.050**	1.92 (1.21, 3.03)	**0.020**	1.31 (0.75, 2.29)	0.425
	No/Unknown	*ref*		*ref*		*ref*		*ref*		*ref*		*ref*	

**Table 3:** Multivariable analysis of receipt of treatment. This table lists the odds ratio with a 90% confidence interval and p-value of receiving surgery, radiation, enrollment in a clinical trial, any form of chemotherapy, cytotoxic chemotherapy, or targeted chemotherapy. Significant p-values are bolded.

		Surgery		Radiation		Clinical Trial		Chemo (any)		Chemo (cytotoxic)		Chemo (targeted)	
Variable		OR (90% CI)	p-value	OR (90% CI)	p-value	OR (90% CI)	p-value	OR (90% CI)	p-value	OR (90% CI)	p-value	OR (90% CI)	p-value
**Gender**	Male	*ref*		*ref*		*ref*		*ref*		*ref*		*ref*	
	Female	0.94 (0.71, 1.24)	0.698	1.01 (0.75, 1.36)	0.967	1.12 (0.86, 1.46)	0.480	0.80 (0.61, 1.05)	0.181	0.77 (0.58, 1.01)	0.114	0.85 (0.61, 1.19)	0.438
	Unknown	0.61 (0.06, 5.95)	0.719	2.08 (0.13, 33.79)	0.666	--	--	380557.3 (0.00, ∞)	0.973	857097.4 (0.00, ∞)	0.972	23.81 (0.91, 620.87)	0.110
**Age**	< 1 year	0.91 (0.48, 1.74)	0.817	0.29 (0.14, 0.63)	**0.008**	0.46 (0.25, 0.85)	**0.039**	0.80 (0.42, 1.54)	0.576	0.95 (0.50, 1.78)	0.884	0.35 (0.13, 0.89)	**0.065**
	1–4 years	*Ref*		*ref*		*ref*		*ref*		*ref*		*ref*	
	5–9 years	1.15 (0.78, 1.68)	0.551	0.91 (0.60, 1.39)	0.720	0.73 (0.51, 1.06)	0.163	0.49 (0.34, 0.71)	**0.002**	0.50 (0.34, 0.73)	**0.003**	0.54 (0.35, 0.84)	**0.021**
	10–14 years	1.62 (1.07, 2.45)	**0.056**	1.14 (0.73, 1.77)	0.633	0.79 (0.54, 1.16)	0.320	0.47 (0.31, 0.70)	**0.002**	0.57 (0.38, 0.85)	**0.021**	0.50 (0.31, 0.80)	**0.016**
	15 + years	2.34 (1.46, 3.75)	**0.003**	1.71 (1.06, 2.76)	**0.064**	0.61 (0.40, 0.94)	**0.060**	0.56 (0.36, 0.87)	**0.030**	0.76 (0.50, 1.18)	0.305	0.50 (0.29, 0.86)	**0.034**
	Unknown	0.61 (0.04, 10.15)	0.772	--	--	--	--	--	--	--	--	--	--
**Ethnicity**	Hispanic	1.14 (0.78, 1.67)	0.566	0.77 (0.52, 1.15)	0.282	0.82 (0.58, 1.17)	0.358	0.88 (0.61, 1.27)	0.580	1.11 (0.78, 1.59)	0.622	0.83 (0.54, 1.27)	0.471
	Non-Hispanic	*Ref*		*ref*		*ref*		*ref*		*ref*		*ref*	
	Unknown	0.97 (0.57, 1.65)	0.920	0.59 (0.33, 1.05)	0.133	0.39 (0.23, 0.65)	**0.003**	0.51 (0.30, 0.87)	**0.039**	0.57 (0.33, 1.00)	**0.097**	0.63 (0.31, 1.30)	0.294
**Race**	White	*ref*		*ref*		*ref*		*ref*		*ref*		*ref*	
	Black	0.80 (0.39, 1.62)	0.598	0.84 (0.37, 1.91)	0.730	0.93 (0.47, 1.84)	0.860	0.81 (0.40, 1.64)	0.626	0.65 (0.31, 1.38)	0.345	0.80 (0.36, 1.80)	0.655
	AI/AN	1.39 (0.44, 4.39)	0.633	0.70 (0.28, 1.76)	0.522	0.53 (0.22, 1.29)	0.243	0.28 (0.10, 0.76)	**0.037**	0.80 (0.32, 1.99)	0.688	0.13 (0.03, 0.57)	**0.023**
	Asian/PI	0.58 (0.24. 1.36)	0.292	0.80 (0.31, 2.03)	0.693	0.47 (0.20, 1.11)	0.148	0.82 (0.34, 1.98)	0.708	1.41 (0.59, 3.37)	0.514	1.16 (0.45, 3.02)	0.795
	Unknown	0.94 (0.59, 1.50)	0.817	1.36 (0.83, 2.23)	0.307	0.40 (0.25, 0.62)	**< .001**	0.57 (0.36, 0.90)	**0.041**	0.65 (0.41, 1.02)	0.118	0.86 (0.48, 1.52)	0.655
**Urban/Rural**	Urban	*ref*		*ref*		*ref*		*ref*		*ref*		*ref*	
	Rural	1.06 (0.75, 1.51)	0.781	0.85 (0.58, 1.24)	0.475	0.89 (0.64, 1.23)	0.553	1.02 (0.73, 1.43)	0.910	0.96 (0.68, 1.36)	0.861	1.38 (0.93, 2.04)	0.179
	Unknown	0.15 (0.07, 0.34)	**< .001**	0.21 (0.07, 0.65)	**0.024**	0.94 (0.41, 2.16)	0.902	0.13 (0.04, 0.39)	**0.002**	0.16 (0.05, 0.51)	**0.010**	--	--
**Year of Diagnosis**	Before 2001	1.59 (0.93, 2.71)	0.156	1.36 (0.74, 2.50)	0.410	2.03 (1.19, 3.44)	**0.028**	2.85 (1.66, 4.88)	**0.001**	3.54 (2.04, 6.13)	**< .001**	0.15 (0.04, 0.54)	**0.014**
	2001–2010	1.69 (1.18, 2.42)	0.016	0.75 (0.52, 1.09)	0.206	2.96 (2.11, 4.16)	**< .001**	1.29 (0.92, 1.83)	0.220	1.38 (0.97, 1.97)	0.131	0.49 (0.32, 0.76)	**0.007**
	2011–2020	*ref*		*ref*		*ref*		*ref*				*ref*	
	Unknown	1.88 (0.10, 34.66)	0.723	544573.4 (0.00,∞)	0.980	72015.50 (0.00, ∞)	0.981	1.441E11 (0.00, ∞)	0.966	7.963E10 (0.00, ∞)	0.967	2056746 (0.00, ∞)	0.982
**Tumor Type**	Low-grade glioma	*ref*		*ref*		*ref*		*ref*		*ref*		*ref*	
	High-grade glioma	0.29 (0.18, 0.47)	**< .001**	7.16 (4.30, 11.93)	**< .001**	1.60 (1.00, 2.55)	**0.100**	2.08 (1.28, 3.38)	**0.014**	1.02 (0.61, 1.69)	0.954	2.67 (1.58, 4.54)	**0.002**
	Other/unspecified glioma	0.24 (0.12, 0.46)	**< .001**	1.53 (0.71, 3.32)	0.361	0.43 (0.20, 0.94)	**0.077**	0.49 (0.23, 1.03)	**0.116**	0.26 (0.09, 0.75)	**0.036**	0.36 (0.12, 1.06)	0.120
	Embryonal tumor	0.56 (0.25, 1.25)	0.236	20.48 (7.58, 55.33)	**< .001**	8.46 (2.84, 25.17)	**0.001**	23.17 (4.06, 132.21)	**0.003**	5.24 (2.24, 12.26)	**0.001**	0.21 (0.07, 0.66)	**0.026**
	Ependymoma	3.64 (1.53, 8.68)	**0.014**	12.89 (6.86, 24.23)	**< .001**	4.09 (2.25, 7.43)	**< .001**	0.84 (0.47, 1.50)	0.631	0.88 (0.49, 1.59)	0.721	0.76 (0.39, 1.49)	0.506
	Germ cell tumors	0.22 (0.12, 0.39)	**< .001**	9.41 (4.99, 17.72)	**< .001**	1.75 (0.94, 3.23)	0.136	13.56 (5.87, 31.33)	**<.001**	6.78 (3.57, 12.87)	**<.001**	0.06 (0.01, 0.39)	**0.012**
	Craniopharyngioma	16.52 (2.85, 95.88)	**0.009**	8.03 (4.11, 15.67)	**< .001**	1.97 (1.03, 3.77)	**0.087**	0.64 (0.31, 1.30)	0.299	0.16 (0.05, 0.56)	**0.017**	1.80 (0.87, 3.72)	0.181
	ATRT	1921423 (0.00, ∞)	0.975	15.48 (5.84, 41.01)	**< .001**	6.97 (2.27, 21.40)	**0.004**	6.32 (1.67, 23.88)	**0.022**	3.14 (1.29, 7.68)	**0.035**	--	0.943
	Meningioma	1.65 (0.44, 6.21)	0.533	5.27 (1.87, 14.85)	**0.008**	0.53 (0.16, 1.74)	0.378	0.80 (0.26, 2.45)	0.744	1.29 (0.42, 3.93)	0.707	--	0.962
	Medulloblastoma	2.86 (1.36, 6.00)	**0.020**	18.60 (9.89, 34.97)	**< .001**	3.70 (2.09, 6.56)	**< .001**	13.53 (5.87, 31.17)	**< .001**	8.44 (4.71, 15.14)	**< .001**	0.16 (0.07, 0.36)	**< .001**
	Choroid plexus	1.23 (0.45, 3.34)	0.731	0.46 (0.12, 1.79)	0.349	0.19 (0.05, 0.69)	**0.034**	0.16 (0.05, 0.50)	**0.009**	0.40 (0.13, 1.23)	0.180	0.13 (0.02, 0.77)	**0.059**
	Unknown	0.47 (0.28, 0.80)	**0.018**	1.48 (0.82, 2.67)	0.270	0.95 (0.56, 1.59)	0.865	1.29 (0.76, 2.17)	0.427	1.33 (0.77, 2.30)	0.395	0.99 (0.52, 1.89)	0.978
**Grade**	Low	*ref*		*ref*		*ref*		*ref*		*ref*		*ref*	
	High	1.29 (0.61, 2.71)	0.571	6.27 (2.93, 13.42)	**< .001**	3.41 (1.81, 6.44)	**0.001**	4.97 (2.37, 10.45)	**<.001**	2.39 (1.25, 4.54)	**0.026**	2.79 (1.38, 5.63)	**0.016**
	Unknown	0.62 (0.35, 1.09)	0.160	3.08 (1.70, 5.57)	**0.002**	3.76 (2.19, 6.45)	**< .001**	1.52 (0.91, 2.51)	0.176	1.09 (0.64, 1.85)	0.793	1.34 (0.74, 2.40)	0.418
**Metastatic Disease**	Present	0.93 (0.45, 1.92)	0.861	1.18 (0.56, 2.45)	0.717	1.40 (0.72, 2.71)	0.409	2.71 (1.17, 6.30)	**0.051**	2.26 (1.17, 4.38)	**0.043**	3.30 (1.58, 6.90)	**0.008**
	Absent	*ref*		*ref*		*ref*		*ref*		*ref*		*ref*	
	Unknown	0.57 (0.36, 0.91)	**0.045**	0.67 (0.43, 1.05)	0.141	0.52 (0.34, 0.79)	**0.010**	0.50 (0.33, 0.76)	**0.007**	0.75 (0.50, 1.14)	0.259	0.68 (0.42, 1.11)	0.194
**Tumor Size**	Less than 6cm	*ref*		*ref*		*ref*		*ref*		*ref*		*ref*	
	6cm or greater	2.55 (0.96, 6.75)	0.114	1.06 (0.52, 2.17)	0.898	0.61 (0.32, 1.19)	0.223	1.10 (0.56, 2.16)	0.813	1.44 (0.74, 2.78)	0.363	1.41 (0.69, 2.89)	0.429
	Unknown	0.76 (0.51, 1.16)	0.286	0.66 (0.43, 1.00)	**0.098**	0.80 (0.55, 1.16)	0.316	1.06 (0.71, 1.57)	0.808	1.01 (0.68, 1.51)	0.956	1.10 (0.71, 1.71)	0.708
**Number of social concerns** *	One	1.17 (0.81, 1.71)	0.484	1.70 (1.15, 2.53)	**0.027**	1.02 (0.72, 1.45)	0.931	1.25 (0.87, 1.78)	0.313	1.02 (0.71, 1.48)	0.913	1.95 (1.29, 2.95)	**0.008**
	Two	1.42 (0.91, 2.20)	0.193	1.49 (0.96, 2.32)	0.133	1.10 (0.74, 1.64)	0.683	1.07 (0.70, 1.62)	0.799	1.32 (0.87, 1.99)	0.272	1.17 (0.71, 1.92)	0.608
	3 or more	1.64 (0.97, 2.76)	0.120	1.94 (1.16, 3.22)	**0.032**	1.66 (1.04, 2.64)	**0.074**	2.97 (1.82, 4.84)	**< .001**	2.57 (1.62, 4.08)	**< .001**	2.75 (1.64, 4.60)	**0.001**
	None/Unknown	*ref*		*ref*		*ref*		*ref*		*ref*		*ref*	

* Sum of insurance, financial, and transportation concerns

**Table 4:** Univariate and multivariable analysis of time from onset of symptoms as noted in the patient’s history to first time receiving medical care. Significant p-values are bolded

		Low Grade				High Grade			
		Time from first symptoms to medical care less than 54 days (ref = greater than 54 days)				Time from first symptoms to medical care less than 31 days (ref = greater than 31 days)			
Variable		OR (90% CI)	p-value	aOR (90% CI)	p-value	OR (90% CI)	p-value	aOR (90% CI)	p-value
**Gender**	Male	*ref*		*ref*		*ref*		*ref*	
	Female	0.87 (0.57, 1.34)	0.593	0.85 (0.53, 1.36)	0.566	0.93 (0.57, 1.53)	0.819	1.26 (0.69, 2.31)	0.528
**Age**	< 1 year	1.82 (0.66, 5.00)	0.328	1.70 (0.53, 5.41)	0.452	2.25 (0.36, 14.23)	0.470	2.72 (0.30, 24.27)	0.453
	1–4 years	*ref*		*ref*		*ref*		*ref*	
	5–9 years	2.96 (1.54, 5.70)	**0.006**	3.36 (1.65, 6.84)	**0.005**	0.28 (0.13, 0.57)	**0.004**	0.31 (0.14, 0.70)	**0.018**
	10–14 years	1.20 (0.64, 2.26)	0.633	1.47 (0.73, 2.94)	0.362	0.49 (0.24, 1.00)	**0.100**	0.41 (0.18, 0.91)	**0.065**
	15 + years	1.22 (0.57, 2.58)	0.670	1.85 (0.81, 4.21)	0.222	0.18 (0.08, 0.39)	**< .001**	0.17 (0.07, 0.41)	**0.001**
**Ethnicity**	Hispanic	1.09 (0.60, 1.99)	0.803	1.11 (0.56, 2.20)	0.804	0.52 (0.30, 0.89)	**0.046**	0.50 (0.25, 0.97)	**0.085**
	Non-Hispanic	*ref*		*ref*		*ref*		*ref*	
	Unknown	0.57 (0.32, 1.02)	0.110	0.56 (0.19, 1.64)	0.377	0.46 (0.17, 1.26)	0.206	0.61 (0.15, 2.48)	0.565
**Race**	White	*ref*		*ref*		*ref*		*ref*	
	Black	8.56 (1.45, 50.53)	**0.047**	6.63 (1.02, 43.33)	**0.097**	0.79 (0.24, 2.61)	0.745	0.64 (0.16, 2.53)	0.595
	AI/AN	1.22 (0.23, 6.46)	0.842	0.81 (0.13, 5.18)	0.854	0.99 (0.32, 3.08)	0.985	0.93 (0.23, 3.81)	0.933
	Asian/PI	1.22 (0.23, 6.46)	0.842	0.87 (0.15, 5.05)	0.900	3.95 (0.64, 24.46)	0.216	7.38 (0.88, 62.04)	0.123
	Unknown	0.83 (0.49, 1.41)	0.560	1.31 (0.52, 3.29)	0.629	0.56 (0.27, 1.18)	0.202	1.08 (0.41, 2.89)	0.892
**Urban/Rural**	Urban	*ref*		*ref*		*ref*		*ref*	
	Rural	1.12 (0.66, 1.90)	0.716	1.12 (0.63, 2.00)	0.753	1.41 (0.78, 2.57)	0.338	1.24 (0.60, 2.58)	0.627
	Unknown	0.61 (0.08, 4.63)	0.687	0.57 (0.07, 4.85)	0.666	67285.95 (0.00, 1.00)	0.967	> 999.99 (< 0.001, > 999.99)	0.992
**Year of diagnosis**	Before 2001	0.70 (0.32, 1.53)	0.455	1.64 (0.58, 4.61)	0.432	179918.8 (0.00, 1.00)	0.963	> 999.99 (< 0.001, > 999.99)	0.982
	2001–2010	0.73 (0.46, 1.15)	0.258	1.16 (0.64, 2.10)	0.674	1.13 (0.67, 1.91)	0.701	2.02 (1.06, 3.84)	**0.072**
	2011–2020	*ref*		*ref*		*ref*		*ref*	
**Metastatic disease**	Present	0.61 (0.16, 2.26)	0.536	0.51 (0.12, 2.25)	0.457	1.25 (0.58, 2.72)	0.629	0.88 (0.35, 2.25)	0.823
	Absent	*ref*		*ref*		*ref*		*ref*	
	Unknown	0.56 (0.35, 0.89)	**0.040**	0.65 (0.36, 1.17)	0.226	0.61 (0.36, 1.04)	0.129	0.42 (0.21, 0.84)	**0.039**
**Tumor Size**	Less than 6cm	*ref*		*ref*		*ref*		*ref*	
	6cm or greater	1.27 (0.50, 3.23)	0.674	1.02 (0.36, 2.87)	0.971	1.89 (0.66, 5.37)	0.318	3.22 (0.92, 11.29)	0.125
	Unknown	0.64 (0.38, 1.09)	0.170	0.66 (0.36, 1.22)	0.269	1.31 (0.73, 2.34)	0.446	2.52 (1.19, 5.33)	**0.043**
**Number of social concerns** *	One	1.33 (0.72, 2.43)	0.442	1.17 (0.60, 2.29)	0.703	1.36 (0.71, 2.62)	0.440	1.83 (0.85, 3.96)	0.198
	Two	1.57 (0.83, 2.97)	0.248	1.31 (0.63, 2.71)	0.541	1.75 (0.89, 3.43)	0.170	1.66 (0.71, 3.85)	0.326
	3 or more	2.62 (1.25, 5.50)	**0.033**	2.71 (1.14, 6.45)	**0.059**	0.52 (0.25, 1.07)	0.138	0.43 (0.18, 1.06)	0.123
	None/Unknown	*ref*		*ref*		*ref*		*ref*	

aOR = adjusted odds ratio; ATRT = atypical teratoid rhabdoid tumors; OR = odds ratio; CI = confidence interval

* sum of insurance, financial, and transportation concerns

**Table 5:** Univariate and multivariable analysis of time from the first medical care for presenting symptoms to diagnosis of a CNS malignancy. Significant p-values are bolded

		Low-grade				High-grade			
		Time from first care to diagnosis less than 5 days (ref = greater than 5 days)				Time from first care to diagnosis less than 3 days (ref = greater than days)			
Variable		OR (90% CI)	p-value	aOR (90% CI)	p-value	OR (90% CI)	p-value	aOR (90% CI)	p-value
**Gender**	Male	*ref*		*ref*		*ref*		*ref*	
	Female	0.90 (0.62, 1.30)	0.635	0.91 (0.59, 1.39)	0.707	0.99 (0.63, 1.55)	0.964	0.94 (0.56, 1.56)	0.839
**Age**	< 1 year	1.12 (0.47, 2.65)	0.836	0.83 (0.30, 2.32)	0.765	1.41 (0.56, 3.53)	0.542	0.94 (0.34, 2.63)	0.924
	1–4 years	*ref*		*ref*		*ref*		*ref*	
	5–9 years	0.73 (0.44, 1.22)	0.310	0.51 (0.28, 0.93)	**0.065**	0.95 (0.51, 1.78)	0.900	0.83 (0.42, 1.64)	0.652
	10–14 years	0.81 (0.49, 1.36)	0.507	0.72 (0.40, 1.30)	0.364	1.26 (0.68, 2.32)	0.540	1.15 (0.58, 2.25)	0.740
	15 + years	0.52 (0.27, 0.99)	**0.097**	0.46 (0.23, 0.95)	**0.079**	0.83 (0.41, 1.69)	0.669	0.65 (0.30, 1.41)	0.360
**Ethnicity**	Hispanic	0.82 (0.47, 1.40)	0.534	0.78 (0.42, 1.43)	0.494	1.21 (0.73, 1.99)	0.533	0.92 (0.51, 1.64)	0.806
	Non-Hispanic	*ref*		*Ref*		*ref*		*ref*	
	Unknown	2.12 (1.31, 3.42)	**0.010**	1.31 (0.53, 3.23)	0.627	0.83 (0.29, 2.33)	0.765	1.20 (0.36, 4.02)	0.808
**Race**	White	*ref*		*Ref*		*ref*		*ref*	
	Black	0.15 (0.03, 0.83)	**0.069**	0.18 (0.03, 1.06)	0.111	0.50 (0.13, 1.96)	0.404	0.35 (0.08, 1.54)	0.243
	AI/AN	0.00 (0.00, 1.00)	0.960	< 0.001 (< 0.001, > 99.99)	0.983	1.50 (0.56, 4.01)	0.498	1.24 (0.43, 3.64)	0.739
	Asian/PI	0.89 (0.21, 3.77)	0.895	1.39 (0.29, 6.60)	0.730	1.20 (0.39, 3.73)	0.791	1.45 (0.42, 4.90)	0.622
	Unknown	1.37 (0.88, 2.12)	0.242	0.52 (0.24, 1.17)	0.183	2.62 (1.21, 5.69)	**0.040**	2.57 (1.07, 6.17)	**0.077**
**Urban/Rural**	Urban	*ref*		*ref*		*ref*		*ref*	
	Rural	1.24 (0.78, 1.97)	0.446	1.21 (0.71, 2.05)	0.562	1.85 (1.09, 3.13)	**0.055**	1.67 (0.93, 3.00)	0.151
	Unknown	13.69 (2.33, 80.43)	**0.015**	5.41 (0.65, 44.90)	0.189	121047.1 (0.00, 1.00)	0.966	> 999.999 (< 0.001, > 99.99)	0.990
**Year of diagnosis**	Before 2001	--	--	7.47 (3.24, 17.21)	**< .0001**	2.58 (0.56, 11.90)	0.307	2.68 (0.51, 14.23)	0.330
	2001–2010	1.50 (0.98, 2.31)	0.118	1.21 (0.72, 2.04)	0.551	1.99 (1.22, 3.24)	**0.020**	2.08 (1.21, 3.60)	**0.027**
	2011–2020	*ref*		*Ref*		*ref*		*ref*	
**Metastatic disease**	Present	0.39 (0.07, 2.34)	0.390	0.22 (0.03, 1.97)	0.256	2.06 (1.05, 4.07)	**0.079**	2.22 (1.05, 4.73)	0.081
	Absent	*ref*		*ref*		*ref*		*ref*	
	Unknown	2.39 (1.56, 3.67)	**< .001**	1.36 (0.80, 2.31)	0.345	1.40 (0.86, 2.28)	0.262	1.28 (0.69, 2.22)	0.548
**Tumor Size**	Less than 6cm	*ref*		*Ref*		*ref*		*ref*	
	6cm or greater	3.59 (1.51, 8.55)	**0.015**	4.31 (1.68, 11.10)	**0.011**	1.55 (0.64, 3.73)	0.416	1.75 (0.66, 4.61)	0.343
	Unknown	2.87 (1.66, 4.97)	**0.002**	2.06 (1.11, 3.81)	**0.054**	1.54 (0.89, 2.68)	0.195	1.49 (0.79, 2.80)	0.302
**Number of social concerns** *	One	0.57 (0.33, 0.97)	**0.082**	0.77 (0.42, 1.41)	0.480	1.24 (0.68, 2.27)	0.554	1.43 (0.73, 2.81)	0.388
	Two	0.77 (0.43, 1.37)	0.452	1.19 (0.61, 2.30)	0.672	1.17 (0.65, 2.11)	0.657	1.46 (0.74, 2.91)	0.363
	3 or more	0.60 (0.31, 1.15)	0.195	1.16 (0.54, 2.49)	0.755	1.50 (0.77, 2.90)	0.314	1.86 (0.85, 4.05)	0.190
	None/Unknown	*ref*		*ref*		*ref*		*ref*	

aOR = adjusted odds ratio; ATRT = atypical teratoid rhabdoid tumors; OR = odds ratio; CI = confidence interval

* sum of insurance, financial, and transportation concerns

**Table 6:** Univariate and multivariable analysis of hazard ratios of survival for variables of interest. Significant p-values are bolded

		Overall survival- all patients (N = 858)			
Variable		HR (90% CI)	P-value	aHR (90% CI)	P-value
**Gender**	Male	*ref*		*ref*	
	Female	1.21 (0.92, 1.60)	0.262	0.75 (0.54, 1.04)	0.145
**Age**	< 1 year	1.09 (0.59, 2.00)	0.823	0.63 (0.31, 1.28)	0.286
	1–4 years	*ref*		*ref*	
	5–9 years	0.55 (0.36, 0.82)	**0.014**	0.70 (0.44, 1.10)	0.195
	10–14 years	0.48 (0.30, 0.77)	**0.012**	0.65 (0.37, 1.14)	0.211
	15 + years	0.62 (0.34, 1.11)	0.176	0.60 (0.29, 1.21)	0.230
**Ethnicity**	Hispanic	1.05 (0.75, 1.48)	0.810	0.69 (0.45, 1.04)	0.139
	Non-Hispanic	*ref*		*ref*	
	Unknown	0.94 (0.61, 1.46)	0.828	1.02 (0.55, 1.89)	0.954
	White	*ref*		*ref*	
**Race**	Black	0.76 (0.33, 1.76)	0.588	0.49 (0.18, 1.34)	0.244
	AI/AN	1.72 (0.81, 3.65)	0.238	0.71 (0.30, 1.68)	0.510
	Asian/PI	1.58 (0.74, 3.36)	0.317	3.59 (1.55, 8.31)	**0.012**
	Unknown	1.15 (0.80, 1.65)	0.530	1.24 (0.74, 2.05)	0.494
**Urban/Rural**	Urban	*ref*		*ref*	
	Rural	1.40 (1.01, 1.95)	**0.092**	1.18 (0.82, 1.69)	0.456
	Unknown	1.73 (0.87, 3.46)	0.191	3.38 (1.48, 7.74)	**0.015**
**Year of Diagnosis**	Before 2001	0.57 (0.33, 0.99)	**0.092**	0.97 (0.46, 2.06)	0.951
	2001–2010	1.05 (0.78, 1.41)	0.796	1.20 (0.83, 1.73)	0.420
	2011–2020	*ref*		*ref*	
**Tumor type**	Low-grade glioma	*ref*		*ref*	
	High-grade glioma	26.52 (17.44, 40.33)	**< .001**	35.63 (20.52, 61.87)	**< .001**
	Other/unspecified glioma	1.57 (0.57, 4.32)	0.464	1.24 (0.43, 3.55)	0.735
	Embryonal tumor	10.22 (5.66, 18.45)	**< .001**	19.07 (8.32, 43.69)	**< .001**
	Ependymoma	2.95 (1.54, 5.66)	**0.006**	2.39 (1.12, 5.12)	**0.059**
	Germ cell tumors	0.91 (0.27, 3.08)	0.902	1.19 (0.30, 4.67)	0.834
	Craniopharyngioma	--	.	--	.
	ATRT	13.83 (7.53, 25.40)	**< .001**	20.76 (9.84, 43.80)	**< .001**
	Meningioma	--	.	--	.
	Medulloblastoma	3.25 (1.83, 5.77)	**< .001**	2.93 (1.45, 5.91)	**0.012**
	Choroid plexus	1.40 (0.26, 7.53)	0.742	1.95 (0.32, 11.95)	0.546
	Unknown	2.79 (1.42, 5.51)	**0.013**	2.27 (0.95, 5.45)	0.122
**Grade**	Low	*ref*		*ref*	
	High	14.01 (5.14, 38.19)	**< .001**	2.55 (0.80, 8.12)	0.185
	Unknown	5.40 (2.06, 14.14)	**0.004**	6.76 (1.82, 25.10)	**0.016**
**Primary Site**	Brainstem	5.33 (3.51, 8.10)	**< .001**	3.75 (2.14, 6.57)	**< .001**
	Hemispheres	1.22 (0.79, 1.90)	0.449	0.55 (0.31, 0.98)	**0.088**
	Meningeal	--	.	--	.
	Pineal	1.02 (0.49, 2.14)	0.967	0.33 (0.12, 0.94)	**0.081**
	Sellar/Suprasellar	0.32 (0.15, 0.66)	**0.010**	0.88 (0.37, 2.06)	0.800
	Spinal cord	1.23 (0.61, 2.47)	0.622	1.15 (0.51, 2.59)	0.773
	PNS	2.22 (0.42, 11.85)	0.433	3.47 (0.52, 23.14)	0.281
	Posterior fossa	*ref*		*ref*	
	Thalamus	1.31 (0.65, 2.62)	0.524	0.63 (0.27, 1.48)	0.370
	Ventricles	1.30 (0.71, 2.38)	0.479	1.16 (0.58, 2.34)	0.728
	Other CNS	1.17 (0.52, 2.59)	0.752	1.37 (0.58, 3.27)	0.548
	Non-CNS	7.49 (2.76, 20.39)	**< .001**	12.70 (3.58, 45.10)	**< .001**
	Unknown	2.06 (0.76, 5.60)	0.233	1.26 (0.42, 3.75)	0.726
**Metastatic disease**	Present	2.05 (1.28, 3.27)	**0.012**	2.39 (1.41, 4.05)	**0.007**
	Absent	*ref*		*ref*	
	Unknown	0.73 (0.53, 0.99)	**0.093**	0.70 (0.45, 1.09)	0.184
**Tumor size**	Less than 6cm	*ref*		*ref*	
	6cm or greater	1.29 (0.73, 2.30)	0.461	1.56 (0.82, 2.98)	0.256
	Unknown	0.82 (0.57, 1.18)	0.380	1.20 (0.78, 1.85)	0.491
**Number of social concerns** *	One	1.36 (0.95, 1.95)	0.153	0.73 (0.49, 1.09)	0.202
	Two	1.04 (0.68, 1.61)	0.867	0.58 (0.36, 0.95)	**0.066**
	3 or more	1.84 (1.24, 2.73)	**0.011**	0.93 (0.57, 1.50)	0.801
	None/Unknown	*ref*		*ref*	

aHR = adjusted hazard ratio; ATRT = atypical teratoid rhabdoid tumors; HR = hazard ratio; CI = confidence interval

* sum of insurance, financial, and transportation concerns

## Data Availability

All analyzed data are presented in the manuscript and supplemental materials.
